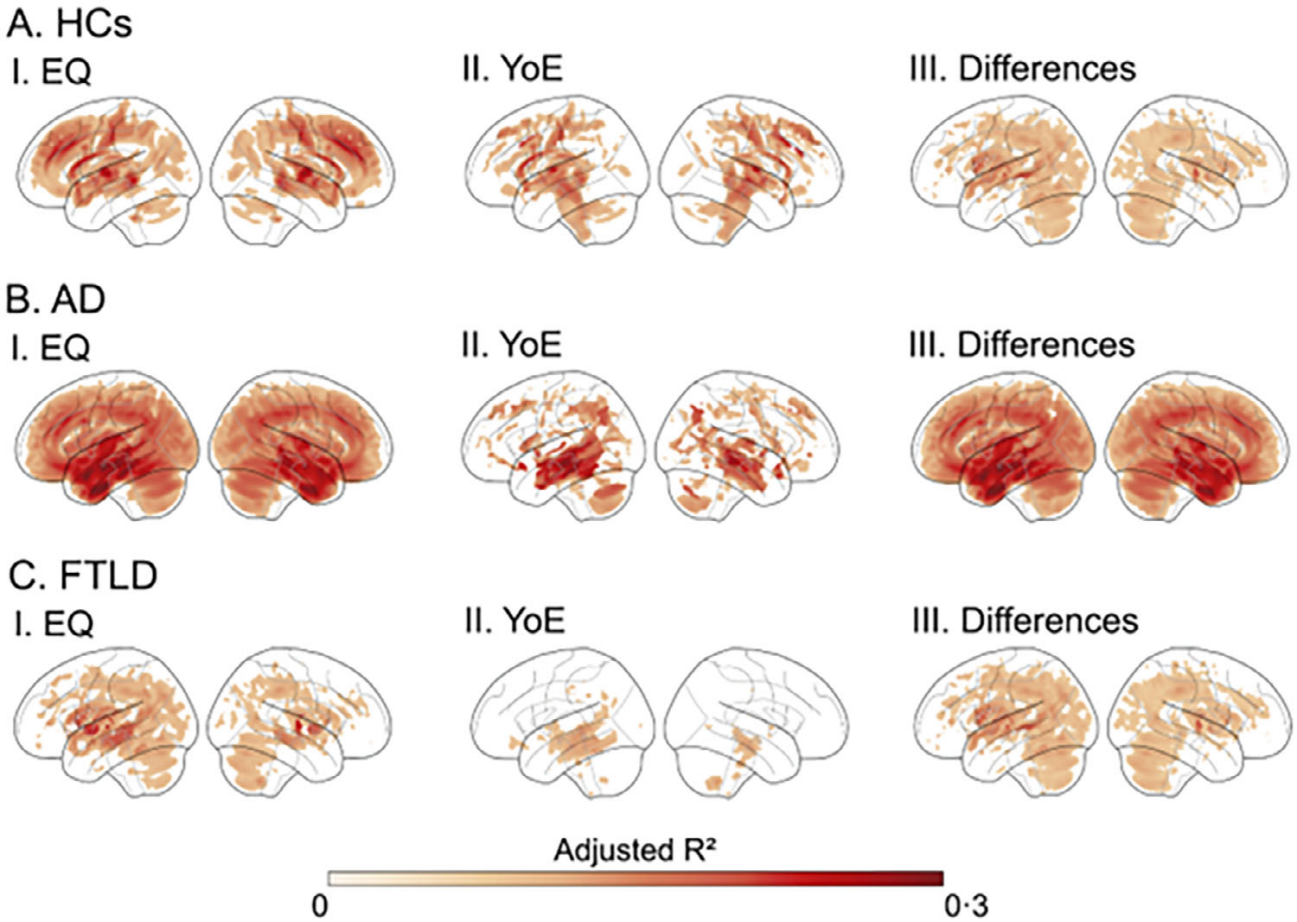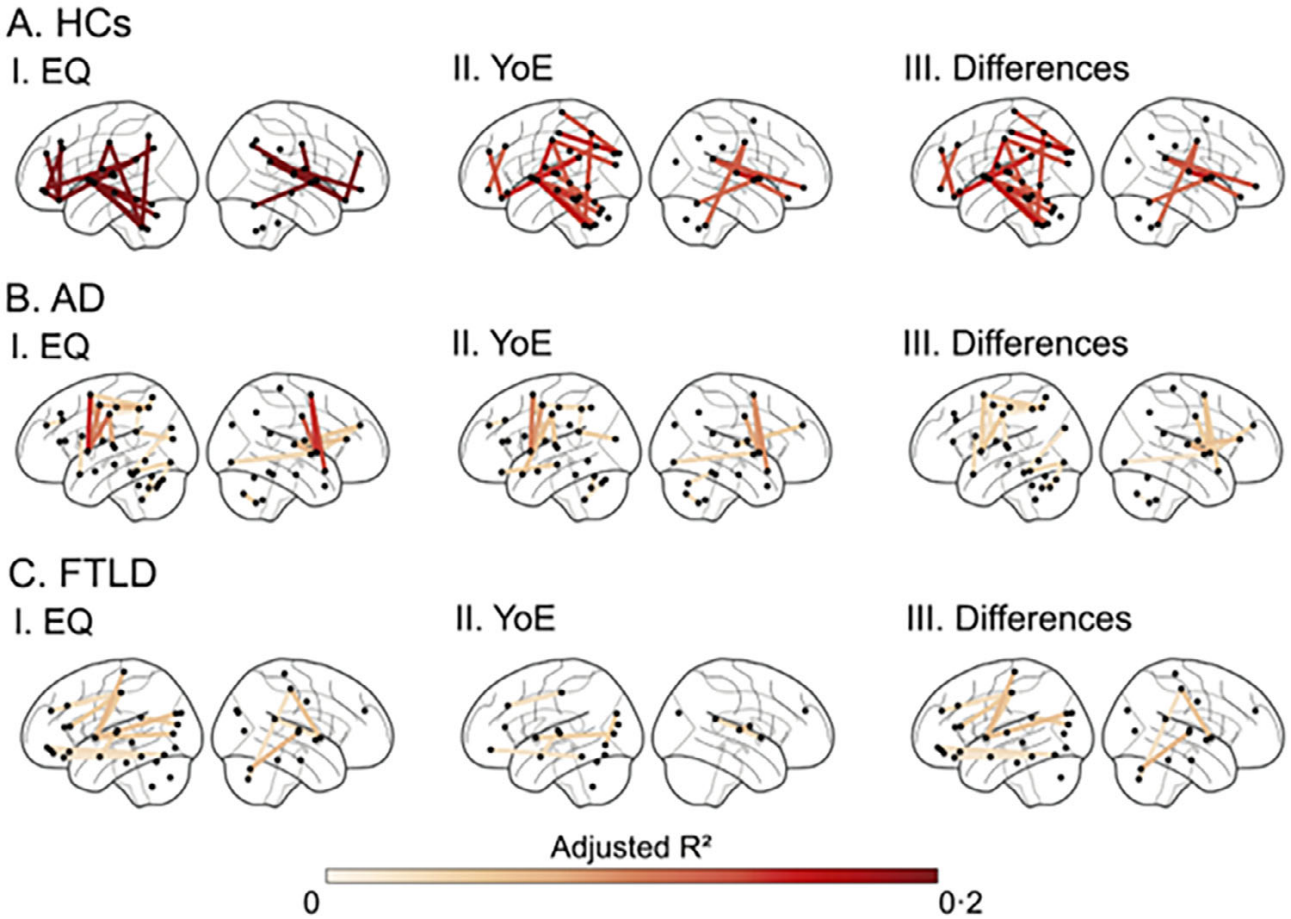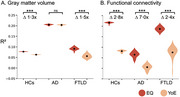# Biological embedding of educational disparities in aging and neurodegeneration across global settings

**DOI:** 10.1002/alz70862_109890

**Published:** 2025-12-23

**Authors:** Raul Gonzalez‐Gomez, Hernan Hernandez, Joaquín Migeot, Josephine Cruzat, Agustina Legaz, Sol Fittipaldi, Marcelo Adrian Maito, Vicente Medel, Enzo Tagliazucchi, Pablo Barttfeld, Daniel Franco O'Byrne, Ana Maria Castro Laguardia, José Alberto Ávila Funes, María Isabel Behrens, Nilton Custodio, Temitope Hannah Farombi, Adolfo M. Garcia, Indira Ruth Garcia Cordero, Maria Eugenia Godoy, Cecilia Gonzalez Campo, Kun Hu, Brian Lawlor, Maira Okada de Oliveira, Stefanie Pina‐Escudero, Katherine L. Possin, Elisa de Paula, França Resende, Pablo A Reyes, Andrea Slachevsky, Leonel Tadao Takada, Victor Valcour, Robert Whelan, Görsev Yener, Jennifer S. Yokoyama, Carlos Coronel‐Oliveros, Agustin Ibanez

**Affiliations:** ^1^ Latin American Brain Health Institute (BrainLat), Universidad Adolfo Ibáñez, Santiago, Región Metropolitana de Santiago Chile; ^2^ Latin American Institute for Brain Health (BrainLat), Universidad Adolfo Ibañez, Santiago Chile; ^3^ Latin American Brain Health Institute (BrainLat), Universidad Adolfo Ibañez, Santiago de Chile, Chile, Santiago Chile; ^4^ Latin American Brain Health Institute (BrainLat), Universidad Adolfo Ibañez, Santiago Chile; ^5^ Global Brain Health Institute, Trinity College Dublin, Dublin Ireland; ^6^ Instituto de Investigaciones Psicológicas (IIPsi, CONICET‐UNC), Facultad de Psicología, Universidad Nacional de Córdoba, Córdoba, Córdoba Argentina; ^7^ Latin American Brain Health Institute (BrainLat), Universidad Adolfo Ibáñez, Santiago, Región Metropolitana Chile; ^8^ Center for Social and Cognitive Neuroscience, Adolfo Ibañez University, Santiago, Región Metropolitana Chile; ^9^ Instituto Nacional de Ciencias Médicas y Nutrición Salvador Zubirán, Mexico City, DF Mexico; ^10^ Centro de Investigación Clínica Avanza (CICA), Hospital Clínico Universidad de Chile, Santiago Chile; ^11^ Hospital Clínico de la Universidad de Chile, Santiago de Chile Chile; ^12^ Clínica Alemana‐Universidad del Desarrollo, Santiago Chile; ^13^ Unit Cognitive Impairment and Dementia Prevention, Peruvian Institute of Neurosciences, Lima, Peru, Lima, Lima Peru; ^14^ Global Brain Health Inistitute, Trinity College Dublin, Dublin, Dublin Ireland; ^15^ Global Brain Health Institute, University of California, San Francisco, CA USA; ^16^ Cognitive Neuroscience Centre, University of San Andres, Victoria, Buenos Aires Argentina; ^17^ Tanz Centre for Research in Neurodegenerative Diseases, University of Toronto, Toronto, ON Canada; ^18^ Cognitive Neuroscience Center (CNC), Universidad de San Andrés, Buenos Aires, Buenos Aires Argentina; ^19^ Massachusetts General Hospital, Boston, MA USA; ^20^ Trinity College Institute of Neuroscience, School of Psychology, Trinity College Dublin, Dublin Ireland; ^21^ Global Brain Health Institute (GBHI), University of California San Francisco (UCSF); & Trinity College Dublin, San Francisco, CA USA; ^22^ Cognitive Neurology and Behavioral Unit (GNCC), University of Sao Paulo, Sao Paulo, Sao Paulo Brazil; ^23^ Global Brain Health Institute, San Francisco, CA USA; ^24^ Memory and Aging Center, Weill Institute for Neurosciences, University of California, San Francisco, San Francisco, CA USA; ^25^ Faculdade de Medicina de Ciências Médicas de Minas Gerais, Belo Horizonte Brazil; ^26^ Instituto de Envejecimiento, Facultad de Medicina, Pontificia Universidad Javeriana, Bogotá, Bogotá Colombia; ^27^ Neurology Service, Department of Medicine, Clínica Alemana, Universidad del Desarrollo, Santiago, Región Metropolitana de Santiago Chile; ^28^ Memory and Neuropsychiatric Clinic (CMYN), Neurology Service, Hospital del Salvador and Faculty of Medicine, University of Chile, Santiago Chile; ^29^ Neuropsychology and Clinical Neuroscience Laboratory (LANNEC), Physiopathology Department ‐ ICBM, Neuroscience and East Neuroscience Departments, Faculty of Medicine, University of Chile, Santiago Chile; ^30^ Geroscience Center for Brain Health and Metabolism (GERO), Santiago Chile; ^31^ Cognitive and Behavioral Neurology Unit, Hospital das Clinicas HCFMUSP, Faculdade de Medicina, Universidade de Sao Paulo, Sao Paulo, Sao Paulo Brazil; ^32^ Memory and Aging Center, University of California San Francisco, San Francisco, CA USA; ^33^ Global Brain Health Institute, University of California, San Francisco, San Francisco, CA USA; ^34^ Trinity College Dublin, Dublin Ireland; ^35^ Global Brain Health Institute (GBHI), Trinity College Dublin, Dublin, Dublin Ireland; ^36^ Dokuz Eylül University, Balçova, Izmir Turkey; ^37^ Izmir University of Economics, Faculty of Medicine, Balçova, Izmir Turkey; ^38^ Brain Dynamics Multidisciplinary Research Center / Dokuz Eylül University, Izmir Turkey; ^39^ University of California, San Francisco, San Francisco, CA USA; ^40^ Memory and Aging Center, Department of Neurology, Weill Institute for Neurosciences, University of California, San Francisco, San Francisco, CA USA; ^41^ Latin American Brain Health Institute (BrainLat), Universidad Adolfo Ibáñez, Santiago Chile; ^42^ Cognitive Neuroscience Center, University of San Andrés, Victoria, Buenos Aires Argentina; ^43^ Global Brain Health Institute (GBHI), University of California San Francisco (UCSF); & Trinity College Dublin, Dublin Ireland

## Abstract

**Background:**

While education is crucial for brain health, evidence mainly relies on individual measures of years of education (YoE), neglecting educational quality (EQ). Whether YoE and EQ have complementary impacts on aging and dementia is unknown.

**Methods:**

We assessed the impact of EQ and YoE on brain health in 7,533 subjects from 20 countries, including healthy controls (HCs), Alzheimer's disease (AD), and frontotemporal lobar degeneration (FTLD). EQ was based on country‐level quality indicators. After applying neuroimage harmonization, we examined their effect on gray matter volume and functional connectivity. Regression models were adjusted for age, sex, and cognition, controlling for multiple comparisons. The impact of image quality was controlled through sensitivity analysis.

**Results:**

Less EQ and YoE were associated with greater brain burden across groups. However, EQ had a stronger impact, mainly targeting the vulnerable areas of each condition. At the whole‐brain level, EQ influenced atrophy (HCs: ∆mean = 2.0 [1.9–2.0] CL95 × 10⁻², *p* < 10⁻⁵; AD: ∆mean = 0.1 [‐0.0–0.3] CL95 × 10⁻², *p* = 0.18; FTLD: ∆mean = 3.5 [3.0–4.0] CL95 × 10⁻², *p* < 10⁻⁵) and networks (HCs: ∆mean = 13.5 [13.2–13.7] CL95 × 10⁻², *p* < 10⁻⁵; AD: ∆mean = 5.9 [5.2–6.7] CL95 × 10⁻², *p* < 10⁻⁵; FTLD: ∆mean = 13.2 [11.2–13.7] CL95 × 10⁻², *p* < 10⁻⁵), 1.3 to 7.0 times more than YoE.

**Conclusion:**

Results support the need to incorporate education quality to study and improve brain health, underscoring the importance of country‐level measures